# Giant Condyloma Acuminata (Buschke-Lowenstein Tumor): Review of an Unusual Disease and Difficult to Manage

**DOI:** 10.1155/2021/9919446

**Published:** 2021-06-30

**Authors:** Jefferson F. Nieves-Condoy, Camilo L. Acuña-Pinzón, José L. Chavarría-Chavira, Diego Hinojosa-Ugarte, Luis A. Zúñiga-Vázquez

**Affiliations:** ^1^Surgery Department, Hospital Regional de Alta Especialidad del Bajío, León, Guanajuato, Mexico; ^2^Oncology Surgery Department, Hospital Regional de Alta Especialidad del Bajío, León, Guanajuato, Mexico

## Abstract

Giant condyloma acuminatum (GCA) or Buschke-Loewenstein tumor is a rare disease, with an estimated prevalence of 0.1%. It was initially described in 1896 by Buschke and later in 1925 by Buschke and Loewenstein. Classic condyloma acuminata (CCA) and squamous cell carcinoma (SCC) were initially described as different entities. These three entities are currently considered to correspond to the same spectrum of different but not exclusive malignant transformations, associated with multiple risk factors such infection by human papilloma virus (HPV), immunodeficiencies, poor hygiene, multiple sexual partners, and chronic genital infections. HPV subtypes 6 and 11 are associated with 90% of GCA. It presents as a cauliflower-like tumor in the genital region with bad odor, bleeding, and local infection, differential diagnosis with multiple conditions should be considered, and sexually transmitted diseases should always be investigated. GCA has a higher rate of malignant transformation than CCA and tends to infiltrate adjacent soft tissues. The therapeutic approach is controversial but is considered that the resection with free edges is the gold standard and can be combined with adjuncts. The recurrence rate is high. Overall mortality is 21% and is associated with morbidity caused by recurrences. Imiquimod cream 5% has recently shown good results as monotherapy and in combination with ablative and surgical treatments. The quality of life is diminished in patients with this condition. In this review, we address the different aspects of this rare entity including the therapeutic approach.

## 1. Introduction

Giant condyloma is a rare entity characterized by an exophytic cauliflower-like growth lesion with tendency to infiltrate adjacent tissues, high rate of recurrence, and potential for malignant transformation [1]. The incidence is little studied. Initially described by Buschke in 1986 and later by Loewenstein in 1925 [2] such a GCA of the penis similar to a tumor but characterized by the absence of histopathological criteria of malignancy. Knoblich and Failing [3] described the clinical and histological similarities and differences between CCA and GCA, the similarities are the orderly proliferation of the epithelium, its thickened, presence of mitotic figures infrequently in the basal and spiny layer and the regular maturation from the basal layer to the stratum corneum showing parakeratosis and hyperkeratosis, however, in GCA was observed, papillomatosis, acanthosis, and elongation of the ridges are more prominent, mitotic activity is more prominent, and keratinization of individual cells and keratin beads can be observed.

GCA also differs from squamous cell carcinoma by an intact basement membrane, preservation of polarity, and absence of lymphatic invasion or metastasis in the lymph nodes. There are authors who do not recognize these differences despite the reports described [4].

GCA is related to several types of HPV, immunosuppression, and less studied risk factors. It has been suggested that CCA and GCA are different entities histologically due to their biological behavior. Currently, it is considered that the CCA, GCA, and SCC correspond to entities of the same continuous but not mandatory spectrum of potentially malignant lesions [5].

There are few series of cases of this disease, and those are compilations of reports of previous cases and analyses of the characteristics described in them, and this limits the knowledge about this entity and the appropriate therapeutic approach. The topical treatments are generally not effective, and surgical treatment should be performed whenever possible [4]. Several therapeutic alternatives and combinations of them have been described in the case reports [6], but due to the infrequency of the disease, a high level of evidence of the results of the different therapeutic regimens is not available. The following review aims to review the literature that describes the characteristics of this disease and to guide the approach to it.

## 2. Epidemiology and Risk Factors

The clinical and histological differences of the CCA and the GCA have been established; however, the cut-off point regarding the size to differentiate them has not been established, being the most important biological behavior with a greater tendency of GCA to recurrence and malignant transformation than CCA [4].

The lack of standardized diagnostic criteria makes it difficult to establish a precise incidence and prevalence of the GCA, reporting several cases in isolation [7]; however, a prevalence of 0.1% of the general population is estimated [8], and an age of presentation between the 4th and 6th decade of life has been suggested [1]. Several cases have also been described in pediatric age range [9].

In the most recent series of cases, a 2.7 : 1 male/female ratio was reported, with an age range of 24 to 77 years, a mean age of 43.9 years (42.9 years in men and 46.6 years in women), and a tendency to present at younger ages [5]. Very few cases have been described in pregnant women [10]. Although it is considered a sexually transmitted disease and sexual abuse should always be suspected when it occurs in pediatric age, cases not associated with sexual transmission have been described, suggesting a mechanism of autoinoculation and heteroinoculation [11].

The main agent related to GCA is the HPV, been associated with HPV subtypes 6 and 11 in more than 90% of cases in patients without other comorbidities and in patients with immunosuppression conditions [12], and cases of multiple subtypes have been reported in the same patient. The association with multiple sexual partners, chronic genital infections, poor hygiene, and immunodeficiencies has been described [13].

Diani et al. [14] reported the aislation of subtypes 6 (low risk HPV) and 52 (high risk HPV) in a 44-year-old man with fast growing GCA who later developed verrucous variant SCC without distant metastasis. He was treated with total penectomy.

The protective role of the vaccine for HPV and related diseases has been reported; however, its role in the treatment or prevention of GCA has not been studied due to the infrequency of the disease, which limits clinical trials; however, it is suggested that it could have some protective role [14].

Several cases associated with immunodeficiency conditions have been described. Gungor et al. [15] reported the case of a 22-year-old smoker woman with type 1 diabetes, who was sexually inactive treated with resection and without recurrence at 12 months of follow-up, and suggests that aggressive local growth could be related to immunosuppression.

Bastola et al. [16] described the case of a 61-year-old man with a history of well-controlled HIV infection who presented with fast growing GCA, a histology report without evidence of neoplasia, and evidence of HPV subtype 16, imaging studies showed deep tissue invasion, and this patient was not a candidate for surgical treatment or radiotherapy due to extensive soft tissue infiltration and multiple fistulas and died of sepsis.

Rachman and Hasan reported the association of systemic lupus erythematosus and GCA with immunosuppressive treatment suggesting the association of immunosuppression and GCA increased risk of infection by HPV [17].

The therapeutic approach in patients with GCA and immunosuppression entails greater complexity that adds to the characteristics of a baseline condition; Li et al. reported a 26-year-old woman with GCA and Netherton syndrome, who was treated with resection and reconstruction with anterolateral thigh flap [18].

## 3. Clinical Presentation

Trombetta and Place [5] described the clinical expression. The main sign described was perineal mass.

Constipation, hemorrhoids, difficulty defecation, difficulty urinating, dysuria, abdominal distension, and fatigue have also been documented [4]. SCC, squamous cell epitheliomas, secondary syphilis, verrucous-vegetative tuberculosis, Nicolas Favre's disease, inguinal granuloma, anogenital amebiasis, and sexual transmitted diseases such as HIV, hepatitis B, and hepatitis C should be systematically ruled out [1].

Imaging studies such as computed tomography and magnetic resonance imaging are necessary to study the local and systemic extension because of the tendency of GCA to infiltrate soft tissues and its high rate of malignant transformation [14, 19].

There are reports of extragenital GCA, which are even more infrequent, prefer the folds, and whose treatment with resection with negative borders is limited by the possibility of formation of postsurgical contractures; Lee et al. [20] reported a case of extragenital GCA in the left axilla in which combined treatment was performed without wide resection to avoid scar contracture.

GCA malignancy can be viewed from two different perspectives. Malignant transformation consists of neoplastic histological confirmation; and malignant behavior consists of the infiltration of adjacent deep tissues regardless of whether there is histological confirmation of malignancy.

Chu et al. [4] in their series reported 42 patients with GCA 56% had histologically confirmed malignancy during follow-up, and of those with malignant behavior at diagnosis 52% had malignant histology. The authors estimate a rate of neoplastic transformation of 56%. On the other hand, Trombetta and Place [5] in their series of 51 patients with GCA reported the presence of neoplasia at the diagnosis of GCA of 58%, of which 8% corresponded to carcinoma in situ and 50% as verrucous carcinoma, SCC, or basal cell carcinoma.

Prasad and Abcarian [21] in their series reported a malignant transformation of anal condyloma acuminata of 1.82% (6/300), 4 were histological and infiltrative, 2 were extensive infiltration of adjacent tissues without histological evidence of malignancy, the difference or association with HPV or its subtypes was not established, and no disseminated malignant disease has documented.

Cases have also been reported in which the association of GCA with cervical intraepithelial neoplasia (CIN) is described [15]. Liu et al. [22] reported the coexistence of GCA with verrucous carcinoma of the vulva and suggested that in the event of a GCA resistant to treatment and a long course of the disease, a biopsy should be performed due to this possibility.

Petrini and Melli [23] have described GCA with infiltration of the female genitalia and subsequent malignant transformation into CIN I and VIN I in a 16-year-old patient.

When SCC occurs in the context of GCA, management must be approached from the context of the patient and sometimes, only palliative measures can be offered to avoid more comorbidities [24].

## 4. Clinical Management

Therapeutic options historically have been topical chemotherapy, intralesional injection of 5-fluoracil (5-FU), interferon, cryotherapy, curettage, CO2 laser vaporization, wide resection alone or with neoadjuvant or adjuvant chemotherapy, chemotherapy alone, radiation therapy, and isolated perfusion [4]. These multiple treatment options, but none of them more effective than the other that has scientific support, demonstrate its poorly established management and high rate of local and regional recurrences. Surgical treatment with negative margins is currently considered the gold standard and should be performed whenever possible [25].

El Bessi et al. have reported the use of neoadjuvant chemotherapy followed by abdominoperineal resection with negative margins and reconstruction in GCA that shows foci of SCC at diagnosis, and this suggests that abdominoperineal resection with or without reconstruction is also considered an option when faced with recurrence [8].

### 4.1. Topical

The application of podophyllin in cases of GCA has poor results; although, it has shown good results in cases of CCA [4].

In the extremely rare cases of extragenital GCA where resection is limited by the functional consequences, the use of combined therapy with topic podophyllotoxin and imiquimod plus cryotherapy weekly has been reported, showing effectiveness and without the functional consequences of surgical alone [20].

Recently, there has been interest in traditional Chinese medicine paiteling in the treatment of CCA and HPV infection; Hu et al. described the efficacy of the traditional Chinese medicine paiteling in the treatment of CCA, in 100 patients with a standardized treatment scheme, without controls, and posttreatment follow-up, and a cure of 92% of the sample and recurrence was reported of 8% at 6 months of treatment [26]. Shu et al. to implement it in a 67-year-old man with AGC on the penis who refused penectomy treatment show a total response without showing recurrence at 3 months of follow-up [27].

### 4.2. Surgery

The gold standard for the treatment of CAG is resection with free margins; however, multiple treatment combinations are described together with surgery both in a neoadjuvant and adjuvant way with different results [6] and without a clear indication of a criterion standardized selection process for the therapeutic approach. A personalized therapeutic approach is recommended in each case [28].

There are several surgical treatment modalities for resection of the GCA, which can be classical surgery and as alternatives like electrocoagulation, radiofrequency, and carbon laser surgery. When opting for surgical treatment, complete resection is important; if the defect is large, reconstruction can be performed at the same surgical time or in a delayed manner [29]. Li et al. [18] described the first reconstruction with ALT in their case report, in which recurrences were treated with topical imiquimod.

Hemper et al. [29] reported a case of a 51-year-old patient with a 10-year evolution of CAG with partial involvement of the external anal sphincter in which they performed resection with preservation of the sphincter and reconstruction of the defect, without recurrence in the follow-up time.

During pregnancy it is suggested to defer surgical management until after delivery because it is associated with spontaneous abortion, intrapartum hemorrhage, preterm delivery, low birth weight. Delivery must be carried out by caesarean section to avoid vertical transmission [10].

### 4.3. Chemotherapy and Radiotherapy

The use of chemotherapy and radiotherapy have been documented in conjunction with surgical resection, both in neoadjuvant and adjuvant forms in GCA with malignant transformation to SCC, protocols based on 5-fluoracil, and mitomycin the first choice [5].

Butler et al. [30] reported neoadjuvant treatment in unresectable GCA with malignant transformation, based on chemotherapy with 5-fluoracil and mitomycin plus radiotherapy with 45 cGy with reduction of the tumor size and improvement of symptoms, followed by abdominoperineal resection 32 weeks after neoadjuvant treatment; at 3 years of follow-up, there was no evidence of recurrence of SCC.

The use of adjuvant chemotherapy with subsequent recurrence, followed by isolated pelvic perfusion with 5-fluoracil, cisplatin, and mitomycin C prior to surgical treatment of recurrence, has been described, reporting a disease-free period at 22 months of follow-up [4].

### 4.4. Imiquimod

In general, topical treatment is considered ineffective due to its high therapeutic failure, but topical imiquimod, an aminoquinolone modifier of the immune response, has shown benefit in the treatment of GCA. Initially, the partial regression of GCA with topical imiquimod plus CO2 laser ablation of the residual lesion was reported, which led to its use in combination with other treatments [31].

The total regression of GCA was described with the use of imiquimod cream 5% cream alone, of a 21-year-old woman, HPV 6 and 11 positives for HIV serology, with application once a day for 12 hours, total regression at 6 months, and without recurrence in a follow-up period of 3 years [31].

Sonthalia et al. [32] described a total regression of penile GCA in a 35-year-old man, with negative HIV serology and evidence of HPV subtype 6. They use imiquimod cream 5%, which was maintained for 12 hours, five days per week for 16 weeks, it was well tolerated, and there was a significant regression at week 12 of treatment, with a recurrence-free period of 5 years.

Although imiquimod has not currently been approved for use in children, there are reports of its use in the treatment of CCA in pediatric age with resolution of the lesions in a shorter time than that reported in adults, without serious adverse effects [9]. Giancristoforo et al. [33] reported the case of a girl with GCA, positive for HPV subtype 6, without sexual abuse story or immunodeficiency, and who was treated with 5% topical imiquimod cream, three times a week, applied for 4 hours, and then the application area was removed and washed, showing considerable reduction after 4 weeks of treatment and complete resolution after 5 months of treatment without sequelae and free recurrence at 2 years of follow-up.

Classically, miquimod cream 5% has been considered a contraindication in mucosal lesions due to the risk of severe inflammation. Recently, Irisawa et al. [33] evaluated the safety and efficacy of imiquimod cream 5% that have in the treatment of intraanal warts, showing a total disappearance of the lesions of 36.8% at 16 weeks of treatment and in 70% at 28 weeks; there were adverse effects in 81% of the participants; however,. there were no serious adverse effects, the most frequent being local erythema, edema, pain, erosion, ulcer, and bleeding.

Recently, Chen et al. [34] reported the use of 5-aminolevulinic photodynamic therapy adjuvant in 2 patients with GCA of the vulva, which was performed once a week, without presenting recurrence at 6 months of follow-up.

## 5. Clinical Evolution

### 5.1. Risk of Recurrence

Chu et al. [4] in their study described an overall recurrence of 67%, approximately 50% of the patients treated with radical surgery as the initial form of treatment had recurrence, these patients had a longer duration of the disease (16 years vs. 10 years) compared to those without recurrence, and the mean time to first recurrence was 10 months. These data suggest an association between the duration of the disease and the initial treatment with risk of recurrence; however, it has the limitation of being a retrospective study based on isolated experiences from previous case reports. Long-term follow-up of GCA is highly recommended due to its high recurrence rate [19].

The morbidity of patients with GCA is associated with soft tissue infiltration and recurrences, including fistulas, abscesses, bleeding from the surgical wound, flap failure, soft tissue infection, urethral obstruction, urinary tract infection, fecal incontinence, and anal stenosis [4].

Overall, mortality is 21%, and they are associated with recurrences and associated morbidities, and histological malignant transformation does not necessarily mean a poor prognosis, since those with histological malignancy have a lower mortality rate (13%) than those without it (33%) [4].

### 5.2. Quality of Life

The series reviewed do not report quality of life surveys associated with this disease nor the impact of treatment or its recurrences and morbidity; however, it is assumed that the disease per se has a negative influence due to the multiple associated symptoms and signs and the morbidity that is associated with the high rate of recurrence, surgical management, chemotherpay, and a greater probability of malignant transformation. Li et al. mentioned in their case report that the signs associated with GCA caused social stigma [18]. And other case reports emphasize the importance that should be given to the emotional sphere [6, 28].

## 6. Conclusions

GCA or Buschke-Lowenstein tumor is a rare disease characterized by a large verrucous tumor, with a high recurrence rate, and it is associated in more than 90% of cases with HPV subtypes 6 and 11; it has a higher malignant transformation index than CCA and is currently considered to correspond to the same malignant transformation spectrum from CCA to SCC, where GCA is the intermediate point. The management is not well established; however, surgical resection with free edges is considered the gold standard, which can be combined with other therapeutic alternatives, and chemotherapy and radiotherapy can be used as neoadjuvant, adjuvant, or salvage measures; overall, mortality is 21% and is associated with complications; imiquimod 5% cream has shown promising results as monotherapy and associated with surgery. The quality of life is diminished.

## References

[1] Ben Kridis W., Werda I., Toumi N. (2019). Buschke — Lowenstein anal tumor: an ambiguous entity. *Experimental Oncology*.

[2] Buschke A., Loewenstein L. (1925). Über Carcinomähnliche Condylomata Acuminata des penis. *Klinische Wochenschrift*.

[3] Knoblich R., Failing J. F. (1967). Giant Condyloma Acuminatum (Buschke-Löwenstein tumor) of the rectum. *American journal of clinical pathology*.

[4] Chu Q. D., Vezeridis M. P., Libbey P. N., Wanebo H. J. (1994). Giant condyloma acuminatum (Buschke-Lowenstein tumor) of the anorectal and perianal regions. *Diseases of the Colon & Rectum*.

[5] Trombetta L. J., Place R. J. (2001). Giant condyloma acuminatum of the anorectum: trends in epidemiology and management. *Diseases of the Colon & Rectum*.

[6] Jorgaqi E., Jafferany M. (2020). Giant condyloma acuminatum (Buschke-Lowenstein tumor): combined treatment with surgery and chemotherapy. *Dermatologic Therapy*.

[7] Kirkilessis G., Karavokyros I. (2020). Giant anal condyloma. *The Pan African Medical Journal,*.

[8] el Bessi M., Dougaz W., Jones M., Jerraya H., Dziri C. (2019). A giant anorectal condyloma is not synonym of malignancy. *Journal of Gastrointestinal Cancer*.

[9] Sikanić Dugić N., Ljubojević Hadžavdić S., Pustišek N., Hiršl Hećej V. (2014). Treatment of anogenital warts in an 18-month-old girl with 5% imiquimod cream. *Acta Dermatovenerologica Croatica*.

[10] Cui T., Huang J., Lv B., Yao Q. (2019). Giant condyloma acuminatum in pregnancy: a case report. *Dermatologic Therapy*.

[11] Elfatoiki F. Z., Hali F., Baghad B., Marnissi F., Chiheb S. (2019). Giant perianal condyloma acuminatum in an infant without sexual abuse. *Archives de Pédiatrie*.

[12] Rydzewska-Rosołowska A., Kakareko K., Kowalik M., Zaręba K., Zbroch E., Hryszko T. (2021). An unexpected giant problem -- Giant condyloma (Buschke-Lowenstein tumor). *International Journal of Infectious disease*.

[13] Bazouti S., Zizi N., Dikhaye S. (2019). Perianal giant condyloma Acuminatum-Buschke-Lowenstein tumor. *La Press Médicale*.

[14] Diani M., Boneschi V., Ramoni S., Gadda F., del Gobbo A., Cusini M. (2015). Rapidly invasive Buschke-Löwenstein tumor associated with human papillomavirus types 6 and 52. *Sexually Transmitted Diseases*.

[15] Gungor Ugurlucan F., Yasa C., Demir O., Yavuz E., Akhan S. E., Dural O. (2019). Giant vulvar condylomata: two cases and a review of the literature. *Case Reports in Obstetrics Gynecology*.

[16] Bastola S., Halalau A., Kc O., Adhikari A. (2018). A gigantic anal mass: Buschke–Löwenstein tumor in a patient with controlled HIV infection with fatal outcome. *Case Report in Infectious Diseases*.

[17] Rachman A., Hasan N. (2016). Giant condyloma acuminata in Indonesian females with SLE under immunosuppressant and steroid therapy. *Case Reports in Immunology*.

[18] Li A. L. K., Walsh S., McKay D. R. (2011). Surgical management of a giant condyloma of Buschke-Lowenstein in a patient with Netherton syndrome using the pedicled anterolateral thigh flap -- a case report. *Journal of Plastic Reconstructive & Aesthetic Surgery*.

[19] Spinu D., Rădulescu A., Bratu O., Checheriţă I. A., Ranetti A. E., Mischianu D. (2014). Giant condyloma acuminatum - Buschke-Lowenstein disease - a literature review. *Chirurgia (Bucur)*.

[20] Lee C., Hsu C., Lee J. Y. (2019). Recalcitrant extragenital giant condyloma acuminatum: A need for combination therapy. *Dermatologic Theraphy*.

[21] Prasad L. M., Abcarian H. (1980). Malignant potential of perianal condyloma acuminatum. *Diseases of the Colon & Rectum*.

[22] Liu G., Li Q., Shang X. (2016). Verrucous carcinoma of the vulva. *Journal of Lower Genital Tract Disease*.

[23] Petrini C., Melli P., Magnani P. (2016). Giant Condyloma (Buschke-Loewenstein tumor) in a 16-year-old patient: case report. *Revista Brasileira de Ginecologia e Obstetrícia*.

[24] Papapanagiotou I. K., Migklis K., Ioannidou G. (2017). Giant condyloma acuminatum-malignant transformation. *Clinical Case Reports*.

[25] Badiu D. C., Manea C. A., Mandu M. (2016). Giant perineal condyloma acuminatum (Buschke-Lowenstein Tumour): a case report. *Chirurgia (Bucur)*.

[26] Hu Y., Lu Y., Qi X. (2019). Clinical efficacy of paiteling in the treatment of condyloma acuminatum infected with different subtypes of HPV. *Dermatologic Theraphy*.

[27] Shu H., Yu B., Li C. (2020). Treatment of giant condyloma acuminatum with paiteling: a case report. *Dermatologic Theraphy*.

[28] Diaz Gómez C. J. (2020). Giant condyloma acuminatum (Buschke–Lowenstein tumor): Combined treatment with surgery and chemotherapy—Further points to be discussed. *Dermatologic therapy 2000*.

[29] Hemper E., Wittau M., Lemke J., Kornmann M., Henne-Bruns D. (2016). Management of a giant perineal condylomata acuminata. *GMS Interdisciplinary Plastic and Reconstructive Surgery DGPW*.

[30] Butler T. W., Gefter J., Kleto D., Shuck E. H., Ruffner B. W. (1987). Squamous-cell carcinoma of the anus in condyloma acuminatum. *Diseases of the Colon & Rectum*.

[31] Combaud V., Verhaeghe C., el Hachem H. (2018). Giant condyloma acuminatum of the vulva: successful management with imiquimod. *JAAD Case Reports*.

[32] Sonthalia S., Gandhi V., Agrawal M., Sharma P. (2019). Successful nonsurgical treatment of penile Buschke–Löwenstein tumor with 12 weeks of 5% imiquimod alone. *International Journal STD AIDS*.

[33] Irisawa R., Tsuboi R., Saito M., Harada K. (2021). Treatment of intra-anal warts with imiquimod 5% cream: a single-center prospective open study. *The Journal of Dermatology*.

[34] Chen X., Zhou Y., Tan Y. (2020). Successful management of giant condyloma acuminatum of vulva with the combination of surgery and photodynamic therapy: report of two cases. *Photodiagnosis and Photodynamic Therapy*.

